# Comparison of Current Regulatory Status for Gene-Based Vaccines in the U.S., Europe and Japan

**DOI:** 10.3390/vaccines3010186

**Published:** 2015-03-18

**Authors:** Yoshikazu Nakayama, Atsushi Aruga

**Affiliations:** Cooperative Major in Advanced Biomedical Sciences, Joint Graduate School of Tokyo Women’s Medical University and Waseda University, 8-1, Kawada-cho, Shinjuku-ku, Tokyo 162-8666, Japan; E-Mail: yoshi-nakayama@fuji.waseda.jp

**Keywords:** regulation, guidelines, plasmid DNA vaccines, viral-vectored vaccines, gene therapy, genetically modified organism

## Abstract

Gene-based vaccines as typified by plasmid DNA vaccines and recombinant viral-vectored vaccines are expected as promising solutions against infectious diseases for which no effective prophylactic vaccines exist such as HIV, dengue virus, Ebola virus and malaria, and for which more improved vaccines are needed such as tuberculosis and influenza virus. Although many preclinical and clinical trials have been conducted to date, no DNA vaccines or recombinant viral-vectored vaccines expressing heterologous antigens for human use have yet been licensed in the U.S., Europe or Japan. In this research, we describe the current regulatory context for gene-based prophylactic vaccines against infectious disease in the U.S., Europe, and Japan. We identify the important considerations, in particular, on the preclinical assessments that would allow these vaccines to proceed to clinical trials, and the differences on the regulatory pathway for the marketing authorization in each region.

## 1. Introduction

Since the first success of the smallpox vaccine in the 18th century, various types of vaccines have been developed: the live-attenuated vaccines, inactivated vaccines, subunit vaccines, toxoid vaccines and conjugate vaccines. However, there is still a long list of infectious diseases for which no effective vaccines exist, such as HIV, dengue virus, Ebola virus and malaria, and for which improved vaccines are needed such as tuberculosis and influenza virus. In view of this, recent vaccine development has concentrated on a new alternative approach, gene-based vaccines such as plasmid DNA vaccines and viral-vectored vaccines [1].

DNA vaccines consist of a bacterial plasmid DNA backbone incorporating the antigen-encoding gene of the pathogens such as the viral envelope region. The plasmid DNA is taken up into the host cell and the antigen is expressed intracellularly. The antigenic protein is processed via the endogenous MHC class I pathway, resulting in the induction of the response of CD8^+^ cytotoxic T lymphocytes (CTLs). Multiple clinical trials have been conducted, but no authorized product for humans exists. More effort is still required for the design of plasmids and delivery method to improve immunogenicity [2,3,4].

As for the viral-vectored vaccines, recent advances in technologies of regulation of viral replication and gene expression have led to the development of novel viral vectors useful for vaccination such as adenovirus, measles virus, poxvirus, and Sendai virus. These viruses are usually genetically modified to deliver and express heterologous antigen gene but are usually modified not to replicate on their own. Like plasmid DNA vaccines, these viral vectors transduce cells that can synthesize the vaccine antigen and induce not only humoral but also CTL immunity [5,6]. The first viral-vectored vaccine expressing heterologous vaccine antigens for humans launched in Australia and Thailand in 2012, but it is not yet authorized in U.S., Europe and Japan [7]. 

In this paper, we provide the current status of the clinical development and the regulations related to the gene-based vaccines intended for use in the prophylaxis of infectious disease in humans in the U.S., Europe and Japan. An overview of gene therapy using plasmid DNA and virus as gene vectors has been reported without distinction of therapeutic and prophylactic purpose [8], and a vaccine-related regulatory overview has also been reported in view of general considerations for conventional vaccines [9]. However, prophylactic vaccines are basically used by healthy people, including children; by contrast, therapeutic products (e.g., treatment of congenital hereditary disease and cancer vaccine) are used by patients already suffering from serious diseases for which no alternative treatment available. Hence, the benefit/risk balance between therapeutic and prophylactic vaccines is clearly different and should be discussed separately.

## 2. Experimental Section

### 2.1. Analysis on Clinical Trials of Gene-Based Prophylactic Vaccines

To review the clinical trials for gene-based vaccines, ClinicalTrial.gov was searched with “vector and vaccine”, “DNA vaccine”, “DNA and vaccine”, “plasmid vaccine”, “genetic vaccine”, “gene vaccine”, “viral vaccine”, “retrovirus and vaccine”, “lentivirus and vaccine”, “vaccinia and vaccine”, “adeno and vaccine”, “adeno-associated and vaccine”, “AAV and vaccine”, “cytomegalovirus and vaccine”, and “Sendai virus and vaccine” as of 7th Nov 2014. We selected only the trials meeting all of the inclusion criteria of interventional trials (*i.e.*, observational trials were excluded); from phase 1 to phase 3 (*i.e.*, phase 0 and 4 trials, and trials lacking data were excluded); using plasmid DNA or recombinant viral expressing the antigen of heterologous infectious pathogen (e.g., vaccinia virus vaccines for smallpox infection which is not expressing the heterologous antigen were excluded); and intending to prophylaxis of infection (*i.e.*, therapeutic or relapse-prophylactic use for the patients were excluded). The selected trials were classified by phase, type of vectors, and target disease. 

### 2.2. Comparison of the Current Regulatory Context for Gene Therapy and Vaccines

We investigated the regulatory context relevant to the development of gene therapy products and vaccines in the U.S., Europe and Japan. First, we examined how gene therapy is defined and how prophylactic and therapeutic gene-based products are classified. Next, we explored the association among guidelines for all types of vaccines, the specific types of vaccines such as plasmid DNA vaccine and viral-vectored vaccine and gene therapy products, and clarified the important considerations for the preclinical assessments before clinical trials. Finally, we compared the regulatory pathway for the marketing authorization of these vaccines and the points of attention for the environmental assessment of medicinal products in each region. 

## 3. Results

### 3.1. Analysis of Clinical Trials of Gene-Based Prophylactic Vaccines

#### 3.1.1. Number of Clinical Trials Registered per Year and Their Phases 

**Figure 1:**
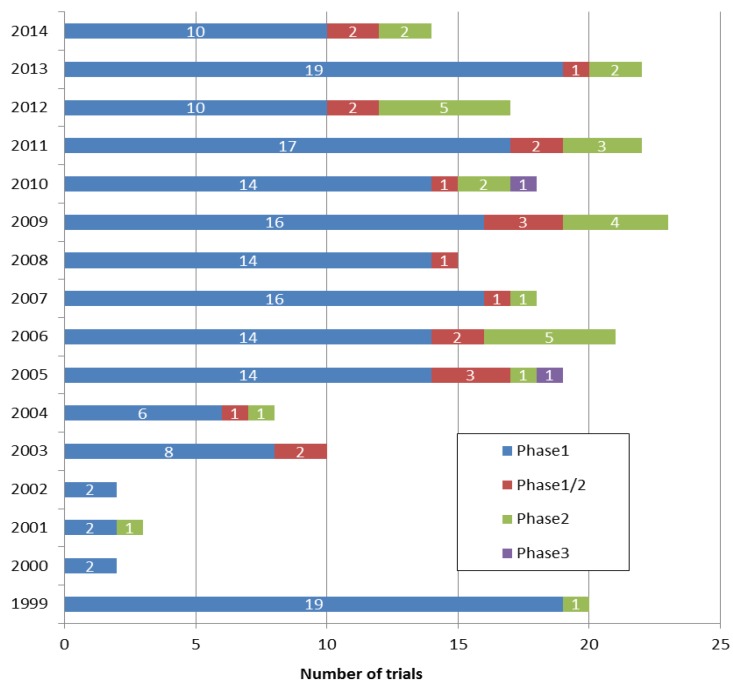
Number of clinical trials registered per year.

ClinicalTrial.gov yielded more than 1200 clinical programs as of November 7, 2014. Many of which did not satisfy our inclusion criteria and 234 clinical trials were extracted definitely. Figure 1 shows number of trials registered per year. ClinicalTrial.gov started registration in 1999 therefore it included the trials which had started before 1999. Since 2005, from 15 to 25 trials are registered per year. More than three quarters of selected clinical trials are phase 1 and they account for 78.2% (183 trials) of all clinical trials. Phase 1/2 and 2 trials make up 9.0% (21 trials) and 12.0% (28 trials) of the total respectively, and phase 3 represent only 1.0% (2 trials) of all trials (Figure 1). Most of the clinical trials are conducted in the U.S. and Europe, and no clinical trial is conducted in Japan. In Japan, single clinical trial for plasmid DNA vaccine on cytomegalovirus-seropositive recipients undergoing allogeneic, hematopoietic cell transplant was registered in ClinicalTrials.gov [1]; however, it does not meet our inclusion criteria (intending to prophylaxis of infection) and was excluded from 234 clinical trials selected finally. 

#### 3.1.2. Diseases Targeted by Prophylactic Gene-Based Vaccines

**Figure 2:**
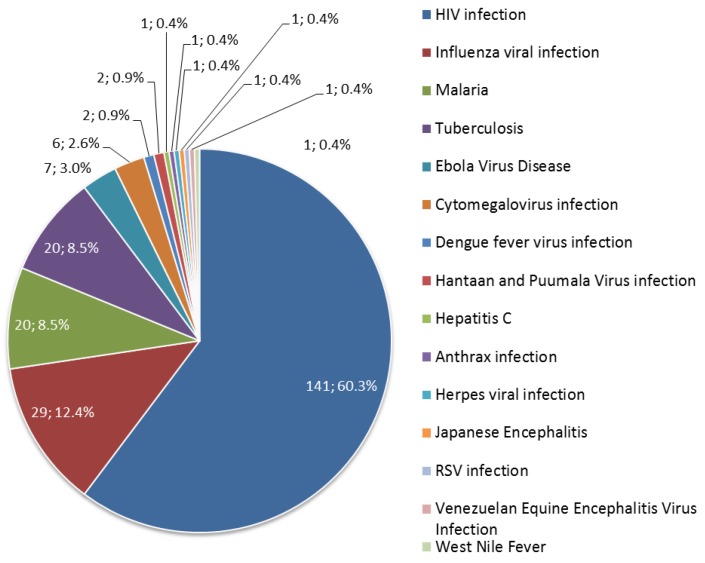
Diseases targeted by prophylactic gene-based vaccines.

Of the 234 clinical trials, the vast majority (141 trials; 60.3%) of clinical trials have addressed HIV infection. Influenza infection, malaria and tuberculosis are the next popular applications for gene-based vaccines, at 12.4% (29 trials), 8.5% (20 trials) and 8.5% (20 trials) of all clinical trials respectively (Figure 2). These infectious diseases own about 90% of all clinical trials. The fifth most common targeted disease is Ebola virus disease and three clinical trials were newly registered in 2014. 

#### 3.1.3. Vectors Used in Prophylactic Gene-Based Vaccines

**Figure 3:**
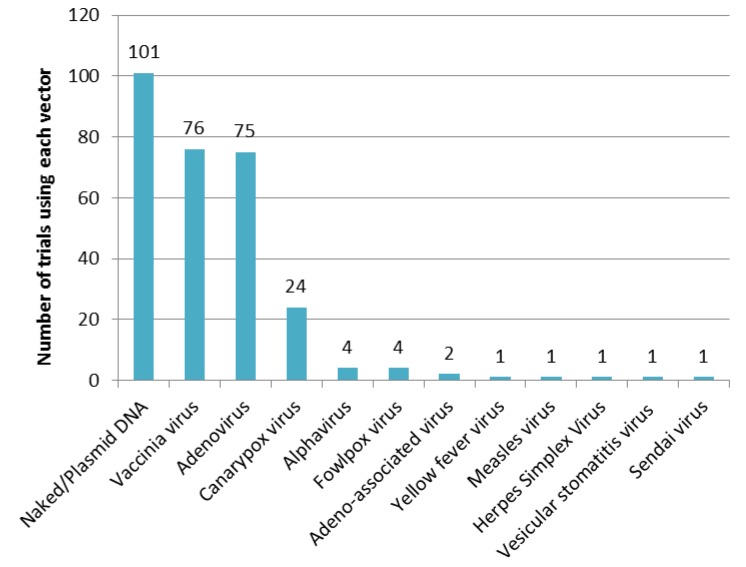
Number of clinical trials using each vector.

Of the 234 clinical trials, naked/plasmid DNA is the most-used as the vaccine antigen vector (101 trials). Next, vaccinia virus (76 trials) and adenovirus (75 trials) are the most often-used vectors (Figure 3). They are often used in combination as prime-boost vaccinations to induce high antibody titres, CD4^+^ T cell frequencies and protective CD8^+^ T cell responses. In about 85% of clinical trials (201 trials), one or more of these three vectors are applied.

### 3.2. Definition of Gene Therapy in the U.S., Europe and Japan

#### 3.2.1. Definition in the U.S.

Regulations in the U.S. are hierarchically composed of “Statutes” (Laws), which are passed by congress and signed by the president, “Regulations” (details of the law), which are written by the FDA and approved by the executive branch, and “Guidance” (FDA’s interpretation of the Regulations) which are legally non-binding, and are written and approved within the FDA. 

**Figure 4:**
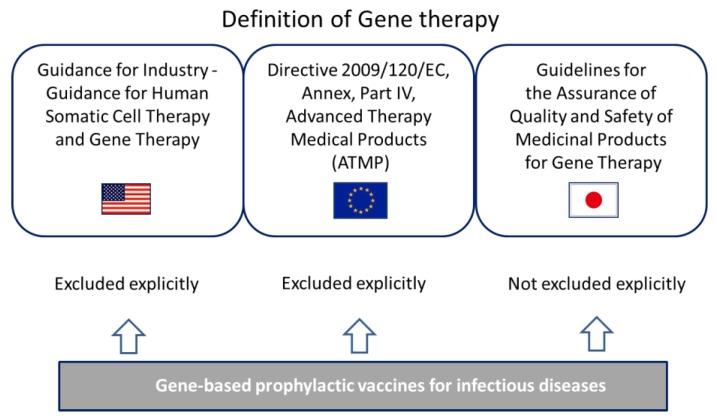
Relationship between gene therapy and gene-based vaccines.

In the guidance for industry entitled “Guidance for human somatic cell therapy and gene therapy” [11] released in 1998 by the center for biologics evaluation and research (CBER), which is the center within the FDA that regulates biological products, gene therapy is defined as “a medical intervention based on modification of the genetic material of living cells”; this publication specifically stipulates that virus or DNA preparations used as preventive vaccines are not covered (Figure 4). In addition, “cells” refer to those modified *ex vivo* for subsequent administration to humans or those given directly to the subject to be altered *in vivo*. Genetic manipulations include those intended for therapeutic or prophylactic purposes, or for cell labeling. 

#### 3.2.2. Definition in the Europe 

In the EU, “Regulation” means a binding legislative act which is applied across the EU, such that each government does not have to take individual action to implement the regulations. “Directives” are also legislative acts although it is up to the individual countries to decide the corresponding action that is needed [12]. “Scientific guidelines” are prepared by the committee for medicinal products for human use (CHMP) within the European medicines agency (EMA) to establish procedures for demonstrating the quality, safety and efficacy of the medicinal products. They do not have legal force; the definitive legal requirements are outlined in the relevant legislative framework such as Directives or Regulations [13].

In the Directive 2009/120/EC [14], amending Directive 2001/83/EC [15] relating to medicinal products for human use as regards advanced therapy medicinal products (ATMP), a gene therapy medicinal product is defined as a biological medicinal product that has the following characteristics: an active substance that contains or consists of a recombinant nucleic acid used in or administered to human beings with a view to regulating, repairing, replacing, adding or deleting a genetic sequence; its therapeutic, prophylactic or diagnostic effect relates directly to the recombinant nucleic acid sequence it contains, or to the product of genetic expression of this sequence. As for vaccines, it is explicitly stated that “vaccines against infectious diseases shall not be included” (Figure 4).

#### 3.2.3. Definition in Japan

In Japan, the act on pharmaceuticals and medical devices (PDA act) amended in November 2014 regulates drugs, medical devices and regenerative medicinal products for human use. In this binding law, gene therapy (not only *ex vivo* but also *in vivo*) is categorized as regenerative medicinal products as well as cellular and tissue-based products. PDA act provides an early approval system (a conditional, time-limited approval system) which only applied to regenerative medicinal products including gene therapy. While prophylactic gene-based vaccines are not excluded explicitly from gene therapy as in the U.S. and Europe, they would be treated separately from therapeutic products in the eye of the law. In a non-binding notification entitled “Guidelines regarding the assurance of quality and safety of drugs for gene therapy” [16] released by the ministry of health, labour and welfare (MHLW), gene therapy is simply defined as “administering genes or cells into which genes are injected for the purpose of the treatment of diseases”. The notification had required that documentation of quality and preclinical information on the drugs for gene therapy should be submitted and reviewed preliminary by the pharmaceutical and medical devices agency (PMDA) before starting clinical trials. While it is not stated explicitly whether prophylactic gene-based vaccines apply the notification, one applicant for clinical trials on a plasmid DNA vaccine has previously filed and completed the preliminary review as well as other gene therapy trials [17]. The Ministry appears to have treated gene-based vaccines as well as gene therapy products (Figure 4) at that time. The mandatory preliminary review was done away with in 2013 to encourage development of gene therapy products; still, applicants are highly recommended to consult with MHLW about how to proceed with the clinical development of gene therapy products and recombinant live vaccines. 

### 3.3. Current Status of Guidelines Specific for the Development of Plasmid DNA Vaccines and Viral-Vectored Vaccines in the U.S., Europe and Japan

#### 3.3.1. Vaccine-Related Guidance in the U.S.

Prophylactic vaccines for infectious diseases including plasmid DNA vaccines and viral-vectored vaccines are classified as biologics and reviewed by the office of vaccines research and review (OVRR) in the CBER of the FDA. Although general guidance for preclinical evaluations has not been released from CBER, the FDA’s approach is summarized in the guidance document entitled “WHO guidelines on nonclinical evaluation of vaccines” published by the WHO in 2003 [18]. Plasmid DNA vaccine and viral-vectored vaccines are within the scope of this guideline. 

Regarding plasmid DNA vaccines, CBER released a specific guidance entitled “Considerations for plasmid DNA vaccines for infectious disease indications” [19] in 2007, which provides key considerations for quality control (manufacture) and preclinical evaluation for plasmid DNA vaccines. Considerations for clinical evaluation are not provided. The CBER had released a former guidance entitled “Points to consider on plasmid DNA vaccines for preventive infectious disease indications” [20] in 1996 based on their review-experiences with other types of conventional vaccines and DNA-based therapeutic products. As the guidance had provided the requirements to initiate phase 1 clinical studies, considerable clinical trials for plasmid DNA vaccine had been conducted. Since knowledge and experience had been accumulated in the CBER, they released a revised guidance in 2007 [21,22] which provides the following considerations in preclinical evaluations:

- studies of immunogenicity
- studies of unintended adverse consequences come from immunomodulatory genes in DNA vaccine
- prime-boost strategies
- assessment whether vaccination causes autoimmune disease
- local reactogenicity and systemic toxicity studies
- biodistribution, persistence, and integration analysis

Regarding viral-vectored vaccines, although guidance for preclinical and clinical evaluation does not exist, that for quality control (manufacture) of live attenuated preparations of viruses, inactivated (killed) whole or subunit virions, purified recombinant proteins, synthetic antigens and live viral vectors expressing specific heterologous vaccine antigens was released from CBER in 2010 [23]. 

#### 3.3.2. Vaccine-Related Guideline in Europe

As a general guideline for preclinical evaluation of vaccines, the committee for proprietary medical products (CPMP) of the EMA released a guideline entitled “Preclinical pharmacological and toxicological testing of vaccines” (CPMP/SWP/465/95) [24] in 1997. All vaccines including live recombinant viral-vectored vaccines are applicable to it. However, plasmid DNA vaccines are explicitly excluded from the scope of the guideline. EMA also released guidance entitled “Note for guidance on the quality, preclinical aspects of gene transfer medicinal products” (CPMP/BWP/3088/99) [25] and “guideline on the non-clinical studies required before first clinical use of gene therapy medicinal products” (EMEA/CHMP/GTWP/125459/2006) [26]. While these guidelines covered plasmid DNA vaccines and viral-vectored vaccine, therapeutic products (e.g., gene therapies, cancer vaccines) and prophylactic products (e.g., vaccines for infection prevention) were discussed without distinction. As noted above, vaccines against infectious diseases were excluded from the definition of gene therapy in 2009, and specific guidelines for gene-based vaccines should have been required.

A specific guideline for viral-vectored vaccines entitled “Guideline on quality, non-clinical and clinical aspects of live recombinant viral-vectored vaccines” (EMA/CHMP/VWP/141697/2009) [27] was released by the CHMP (the successor to CPMP) in 2010 as a supplemental to these guidelines. This guideline provides considerations of quality, preclinical evaluation and clinical evaluation of viral-vectored vaccines intended for use in the prophylaxis of infectious disease in humans. The contents of preclinical evaluation are as follows:

- General considerations
- Pharmacodynamic studies (protection and immunogenicity), Pre-existing immunity
- Non-clinical safety studies (toxicity testing)
- Single and repeated dose toxicity
- Distribution studies
- Reproduction and developmental toxicity studies
- Local tolerance

Regarding plasmid DNA vaccines, a specific guideline has not yet released by EMA but only a concept paper which introduced a direction of the preparation of the guideline is available [28]. Just like FDA, the former guideline [25] was prepared based on hypothetical considerations and required an excessive amount of preclinical investigations such as reproductive/developmental toxicity, genotoxicity/carcinogenicity, pharmacokinetics and potential for chromosomal integration. As knowledge and experience at preclinical and clinical level have been accumulated in the EMA, updated guidance for the use of plasmid DNA as vaccines will be released in the near future and contribute further development of the vaccines. 

#### 3.3.3. Vaccine-Related Guideline in Japan

In Japan, the general non-binding guideline entitled “Guideline for non-clinical studies of preventive vaccines for infectious diseases” [29] and “Guideline for clinical studies of preventive vaccines for infectious diseases” [30] were released in 2010. This guideline explicitly excludes plasmid DNA vaccines and viral-vectored vaccines from its scope so the specific guidelines for these gene-based vaccines do not exist in Japan. In addition, ICH S6 (R1) entitled “Preclinical safety evaluation of biotechnology-derived pharmaceuticals” [31] also does not apply to viral vaccines, DNA vaccines or gene therapy products.

As noted above, a notification for gene therapy [16] exists in Japan. Although it is not stated explicitly whether the prophylactic gene-based vaccines apply, one applicant developing a plasmid DNA vaccine underwent the preliminary review required for gene therapy products by MHLW, setting a precedent for the applicant who plans to start clinical trials for gene-based vaccines. In concrete terms, the following preclinical assessments are required for the optional preliminary review:

- Possibility of the proliferative viral expression
- Cellular cytotoxicity
- Genetic integration with chromosomes
- Safety caused by abnormal expression of expression product
- Carcinogenicity
- Immunogenicity

Table 1 shows the summary of the regulatory context for various kinds of vaccines in the U.S., Europe and Japan. In addition, guidelines released from WHO are included in the table. 

### 3.4. Regulatory Pathway from Clinical Trials to the Marketing Authorization in Each Region

**Figure 5:**
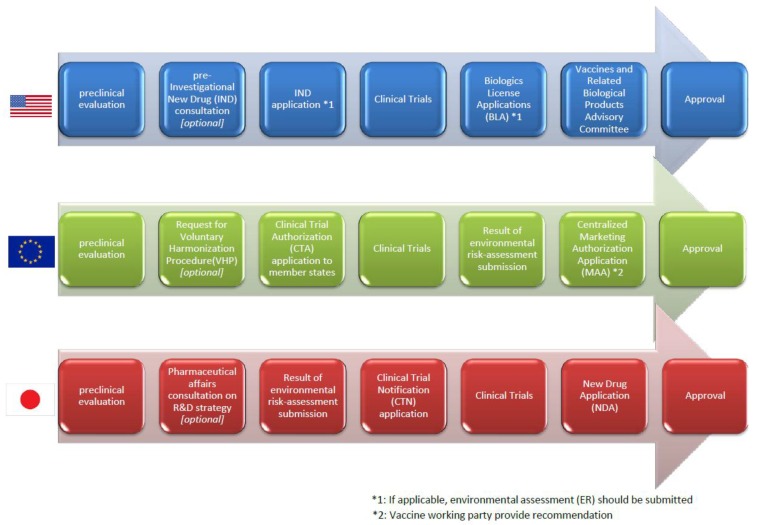
Simplified regulatory pathway from preclinical to the marketing authorization.

In the U.S., an applicant who plans to start clinical trials has to submit an application for the clinical trial, an Investigational New Drug application (IND), which provides the information to assess whether the product is reasonably safe for initial testing in humans by FDA, the manufacturing, the result of animal pharmacology and toxicology studies, and clinical protocols and investigator information. If applicable, the applicant may be able to consult with the FDA as Pre-IND consultation in which the FDA advise the requirements for the IND [32,33]. Then, phase I studies are carried out to assess safety and immunogenicity in a small number of subjects, phase II studies to assess the dosage and the vaccination schedule and phase III studies to verify the protective efficacy of the vaccine in a large number of subjects are typically conducted. After the success of these clinical trials, the applicant can submit a biologics license application (BLA) to the FDA and the application is reviewed by the multidisciplinary FDA reviewer team (*i.e.*, medical officers, microbiologists, chemists, biostatisticians) to make a risk/benefit assessment lead to the recommendation or opposition of the approval of a vaccine. Following the review, the sponsor and the FDA present their findings to the vaccines and related biological products advisory committee (VRBPAC) consists of a non-FDA expert (*i.e.*, scientists, physicians, biostatisticians and a consumer representative) who provides advice to the FDA regarding the safety and efficacy of the vaccine for the proposed indication [34] (Figure 5).

**Table 1:** Regulatory contexts for various kinds of vaccines.

Regulatory agency	Conventional Vaccines *	Plasmid DNA Vaccines-Specific Guidelines	Viral-Vectored Vaccine Specific Guidelines
FDA (U.S.)	None	Considerations for plasmid DNA vaccines for infectious disease indications (2007)	Characterization and qualification of cell substrates and other biological materials used in the production of viral vaccines for infectious disease indications (2010)
		*Contents:* *Quality* *and* *Preclinical* *considerations*	
			*Contents:* *Quality* *c* *onsiderations*
EMA (EU)	Note for guidance on preclinical pharmacological and toxicological testing of vaccines (1995)	None	Guideline on quality, non-clinical and clinical aspects of live recombinant viral vectored vaccines (2010)
	*Applicable for viral-vectored vaccine but DNA vaccine* *is out of* *scope*		*Contents:* *Quality,* *Preclinical* *and* *Clinical* *considerations*
MHLW (Japan)	Guideline for non-clinical studies of vaccines for preventing infectious diseases (2010)	None	None
	*Plasmid DNA vaccine and* *viral-vectored vaccine* *are out of scope*		
WHO	WHO guideline on nonclinical evaluation of vaccines (2003)	Guidelines for assuring the quality and preclinical safety evaluation of DNA vaccines. WHO technical report series No 941, (2007)	None
	*Applicable for* *plasmid DNA vaccine and* *viral-vectored* *vaccine*	*Contents:* *Quality* *and* *preclinical* *considerations*	

* Listed preclinical related guidelines only. Each authority has released several guidelines for specific topics.

In Europe, the clinical trial authorization is granted by each competent authority rather than the EMA. However, as Directive 2001/20/EC came into force with the objective of harmonizing clinical trial processes, a voluntary harmonization procedure (VHP) that allows a single application of multinational clinical trial and the application is evaluated in a single procedure jointly by the competent authorities of EU member states where the multinational clinical trials being planned. This harmonization enables innovative medicines such as gene-based vaccines to be delivered to patients as quickly as possible [35]. Regarding the marketing authorization process of medicinal products, there is also a centralized procedure released from the CHMP, and the gene-based vaccines are applied as one of recombinant DNA technology-derived medicinal products to which the procedure is subject mandatorily. In this procedure, a single marketing authorization application (MAA), a single evaluation and a single marketing authorization which is valid in all member states of the European community are allowed [36]. The vaccine working party (VWP) established by CHMP provides recommendations to them regarding several matters, including MAA (Figure 5).

In Japan, the applicant who plans to start clinical trials has to submit a clinical trial notification (CTN) to the PMDA by each trial. The applicant provides relevant documents: a statement regarding the reason why the sponsoring of the proposed clinical trial is scientifically justified, a protocol of the proposed clinical trial, an explanation document used for informed consent and consent form, and an investigator’s brochure. PMDA conducts a scientific review and make inquiries to the applicant within 30 days, if the CTN is first-time submission. In this period, PMDA and the applicant have to resolve all the inquiries, and the applicant cannot start clinical trials [37]. Before submission of CTN, as noted in Section 3.2.3 and Section 3.3.3, the applicant should have a “pharmaceutical affairs consultation on R&D strategy”, a consultation with PMDA, which is not mandatory but highly recommended [16]. This consultation will help PMDA and the applicant to find any critical issues/inquiries in advance which are difficult to solve within 30 days. After the success of clinical trials, the applicant can submit a new drug application (NDA) to the PMDA and all the data is reviewed by the multidisciplinary PMDA reviewer (*i.e.*, medicine, pharmacy, chemistry, biostatisticians), then the first council with a PMDA reviewer and non-PMDA expert are held. If needed, the applicant presents to the PMDA reviewers and external experts in interview-based board of review. Following second council with PMDA reviewer and non-PMDA expert leading to the decision of recommendation or opposition of the approval of a vaccine, MHLW finally gives approval (Figure 5).

### 3.5. Assessment of the Environmental Impact of Medicinal Products

In parallel with the rapid expansion of modern biotechnology, public concerns about potential adverse effects of the modern biotechnology on the biological diversity are growing.

In Europe, the applicant of a medicinal product containing or consisting of genetically modified organisms (GMOs) such as recombinant virus has to evaluate the potential environmental risks posed by them. Directive 2001/18/EC [38] and Regulation (EC) 726/2004 [39] require the environmental risk assessment (ERA) to be submitted as part of the marketing authorization application (MAA) of the medicinal product. The procedure for the ERA and the information required are stated in the guideline entitled “Environmental risk assessments for medicinal products containing, or consisting of, genetically modified organisms (GMOs)” [40]. As there are procedural and scientific complexities associated with the ERA evaluation, it is recommended that the applicants should request pre-submission meetings to EMA from six months to one year in advance of submission of MAA (Figure 5).

In the U.S., CBER has released draft guidance for industry entitled “Determining the need for and content of environmental assessments for gene therapies, vectored vaccines, and related recombinant viral or microbial products” in 2014 [41], that provides recommendations as to considerations when assessing whether to submit an Environmental Assessment (EA) for biologics. Preparation of EA is required by the proposed action to FDA (e.g., INDs, BLAs, and supplements to BLAs) unless which is qualified as “categorical exclusion”. Although IND is ordinarily categorically excluded from the requirement to submit an EA, the proposed actions to FDA are usually qualified as categorical exclusion on a case-by-case basis in view of whether the actions may affects the quality of the environment significantly. As for BLA, the FDA determine the necessity of EA in view of whether the substances occur naturally in the environment and the approval and license leads to significant alteration on the concentration or distribution of the substance, its metabolites, or degradation products in the environment (Figure 5).

In Japan, there is a law entitled “Act on the conservation and sustainable use of biological diversity through regulations on the use of living modified organisms (LMO)” [42]. Pursuant to the act and regulations under it, the applicant who plans to start clinical trials for pharmaceuticals containing LMO (e.g., viral-vectored vaccines) has to stipulate a rule about the approach in the use of the pharmaceuticals and submit a “Biological diversity risk assessment report” which contains the following information: recipient organism (e.g., taxonomical position and state of distribution in natural environment, history of use, physiological and ecological properties), preparation of LMO (e.g., donor nucleic acid, vector, method of preparation, state of existence of nucleic acid transferred in cells and stability of expression of traits, methods of detection and identification of LMO and their sensitivity and reliability, and difference from the wildlife), and the approach in the use of LMO (e.g., emergency measures be taken to prevent adverse effect on biological diversity) [43]. The applicant has to keep in mind that it often takes about half a year to be approved the use of LMO by regulatory agency (Figure 5). 

## 4. Discussion

The number of clinical studies rose in 2005 and has remained at a level of 15 to 25 trials per year. Phase 3 trials are strictly limited in number, which suggests the difficulty of the induction of desirable immunity by gene-based vaccines. As noted above, combinations of plasmid DNA/viral-vectored vaccines or multiple kinds of vaccines are commonly investigated today. If the optimal combination were developed, the number of Phase 3 pivotal clinical studies to verify the protective efficacy would increase. With respect to the vector selection, retrovirus vectors which are widely used in gene therapy [8] are never used in the vaccine field, and vaccinia viruses, which had been used as smallpox vaccines are used as vectors expressing heterologous antigens in a number of clinical trials. 

We showed that gene-based prophylactic vaccines for infectious diseases are not included in the definition of gene therapy in the U.S. and Europe, and the FDA and EMA have released guidelines specific to the plasmid DNA vaccines and viral-vectored vaccines respectively. They released these guidelines and required safety data including potential risk from their experiences of review of other type of products (e.g., gene therapies, conventional vaccines) first, and revised the guidelines with their knowledge and experience of review of gene-based vaccines, if needed. In Japan, by contrast, MHLW has not released gene-based vaccine-specific guidelines yet and the applicant of clinical trials for gene-based vaccine is recommended to consult with PMDA in the same pathway with gene therapy products at this time.

By comparing the preclinical requirements for the treatment drugs, conventional vaccines and gene-based vaccines, we can find some differences. In ICH M3 (R2) [44], a guidance on nonclinical safety studies for pharmaceuticals, pharmacology studies, general toxicity studies, toxicokinetic and pharmacokinetic studies, reproduction toxicity studies, and genotoxicity studies are introduced as general nonclinical safety studies to support human clinical trials. In addition, an assessment of carcinogenic potential is recommended for drugs that have special cause for concern or are intended for a long duration of use. By contrast, guidelines on the preclinical evaluation for conventional vaccines released by EMA [24] and MHLW [29] as well as WHO [18] state that genotoxicity studies, carcinogenicity studies and pharmacokinetic studies are normally not needed in light of the properties of conventional vaccines. In the guideline for gene-based vaccines such as plasmid DNA vaccines [19] and viral-vectored vaccines [27], these exemptions stated in the guidelines for conventional vaccines would not be applied. For example, the investigation of DNA integration is commonly a concern of health authorities in each region because gene-based vaccines have the risk of tumorigenesis if insertion of encoded DNA into host DNA reduces the activity of a tumor suppressor or increases the activity of an oncogene. Pharmacokinetic studies of viral-vectored vaccines would be important for environmental risk assessments.

We would emphasize that there are some considerations which are not adequately stated in the Japanese gene therapy guideline and therefore that would limit gene-based vaccines to be applied to the guideline without qualification. Regarding DNA vaccines, points worthy of special mention in the FDA guidance are the considerations for autoimmunity and integration analysis. The FDA no longer recommends preclinical assessment as to whether plasmid DNA vaccines cause autoimmune disease, suggesting that the careful monitoring of the general welfare of animals in preclinical immunogenicity and toxicity studies should be enough. It is also stated that integration studies are not needed except when the plasmid persists in any tissue of animals at levels exceeding 30,000 copies per microgram of host DNA by the study’s end, because the risk of integration would be extremely low when levels are below this threshold. Regarding viral-vectored vaccines, a point worthy of special mention in the EMA guidance is the considerations for the assessment of pre-existing immunity, as it might influence the outcome of non-clinical and clinical studies as typified by the clinical trial for HIV vaccine [45].

## 5. Conclusions

Through our study, we clarified some differences in current regulatory condition in each region. As for the gene-based vaccines, as contrasted with gene therapy which is recently provided an early approval system (a conditional, time-limited approval system), undeveloped condition in guideline development is found in particular in Japan. It should be improved to activate clinical trials for gene-based vaccine and to allow Japan to participate in the multinational clinical trials. Further, we hope that guidelines for gene-based vaccines would be harmonized in the U.S., Europe and Japan in the near future and would be kept up-to-date in parallel with the progress in science and technology for the contribution to global public health.
